# Combined bilateral femoral head necrosis and pertrochanteric fracture: a case report

**DOI:** 10.1186/1752-1947-9-25

**Published:** 2015-01-13

**Authors:** Bogdan Deleanu, Radu Prejbeanu, Dan Crisan, Dinu Vermesan, Vlad Predescu, Eleftherios Tsiridis

**Affiliations:** 16 1st Orthopedics and Traumatology Clinic, Emergency Clinical County Hospital Timisoara, 10 I. Bulbuca Blvd, 300737 Timisoara, Romania; 17 ‘Victor Babes’ University of Medicine and Pharmacy Timisoara, 2 E. Murgu Sq., 300041 Timisoara, Romania; 18 ‘St. Pantelimon’ Clinical Emergency Hospital, 340-342 Pantelimon Road, 021659 Bucuresti, Romania; 19 ‘Carol Davila’ University of Medicine and Pharmacy Bucharest, 37 Dionisie Lupu St., 020022 Bucuresti, Romania; 20 Aristotle University Medical School, 54124 Thessaloniki, Greece

**Keywords:** Modular, Bilateral, Primary hip replacement, Hip osteoarthritis, Trochanteric fracture

## Abstract

**Introduction:**

Modular femoral implants have become a regular feature of revision hip surgery. However, for a primary hip arthroplasty, such as a femoral neck fracture case, the implant of choice is a standard femoral component, while compelling literature evidence have made osteosynthesis the standard procedure for the vast majority of trochanteric fractures.

**Case presentation:**

We present the case of a 66-year-old Caucasian woman presenting with two trochanteric fractures associated with primary and secondary hip osteoarthritis that were treated with an uncemented total hip replacement with a modular femoral component.

**Conclusions:**

We found that a modular femoral component can address the issues of stability and, in our case, proved to be a viable solution for treating cases that are complicated by concomitant acetabular or femoral head and neck pathology.

## Introduction

Primary total hip arthroplasty (THA) with a modular femoral component is described in literature as an arthroplasty solution for patients with extensive trochanteric and subtrochanteric fractures associated with primary or secondary hip osteoarthritis (OA) or other acetabular defects that need to be addressed at the time of the surgery, making a sliding hip screw (SHS) or cephalomedullary device (CMD) osteosynthesis undesirable [1]. Furthermore it has been suggested that patients who are at a high risk of implant failure undergo primary hip arthroplasty (cemented or uncemented) for trochanteric hip fractures [2, 3].

## Case presentation

**Figure 1:**
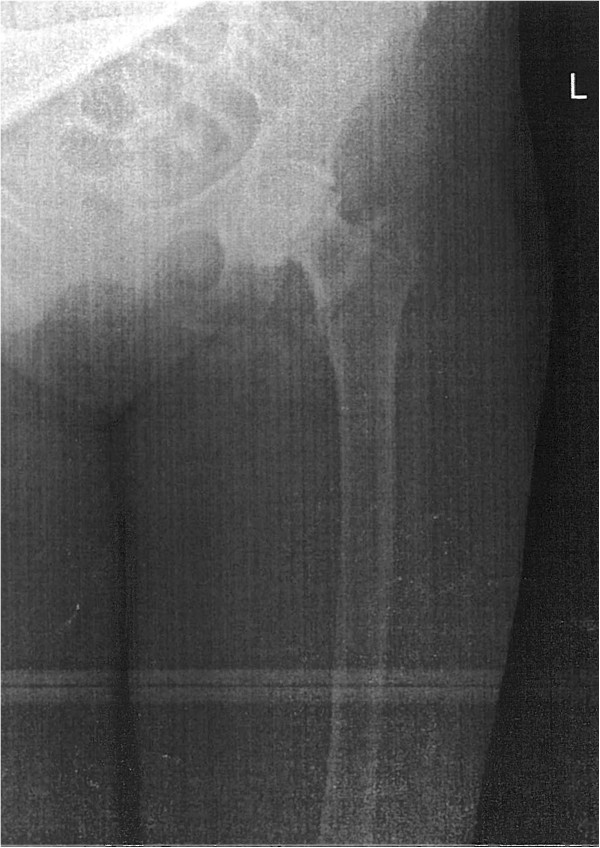
**Emergency X-ray showing an Evans III pertrochanteric fracture and Kellgren-Lawrence III hip osteoarthritis.**

We present the case of a 66-year-old Caucasian woman who presented with a left pertrochanteric fracture caused by a fall from the same level. Her standard emergency antero-posterior (AP) radiographic examination revealed that the fracture was associated with primary hip OA (Figure 1). Because of the pathological combination (the distal extension of the trochanteric fracture beyond the supporting area of a primary femoral stem) and in accordance with previous publications [4–7] the decision was made to treat her with a uncemented modular femoral component with diaphyseal support (Revitan^®^ Straight, Zimmer, Warsaw IN), while the acetabular component was a primary uncemented metal backed polyethylene component (Trilogy^®^ Acetabular System, Zimmer, Warsaw IN).

**Figure 2:**
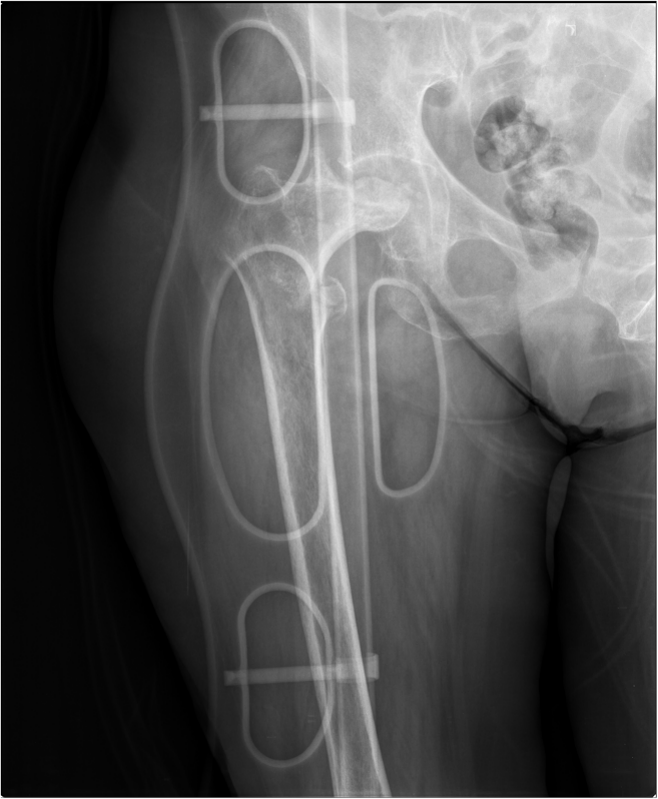
**Emergency X-ray of her right hip showing a simple intertrochanteric fracture associated with femoral head stage VI avascular necrosis, with the femoral head impacted in the acetabular rim.**

At her one-year follow-up visit she had no pain on her left hip, with the expected range of motion (ROM); flexion of 105°; abduction 32°. However, she had intense pain in her right hip associated with a progressing limp for the past five months. Her X-ray revealed stage VI femoral head avascular necrosis (AVN), with the proximal migration of her femoral head with secondary acetabular rim destruction. She was scheduled for a primary THA. A few days after the visit she sustained a fall from the same level that resulted in extreme pain over her already painful right hip, and pain and deformation of the normal anatomy of her right arm. She presented to our emergency room and was diagnosed with a right pertrochanteric fracture (Figure 2) and a wedge-type humerus shaft fracture.

**Figure 3:**
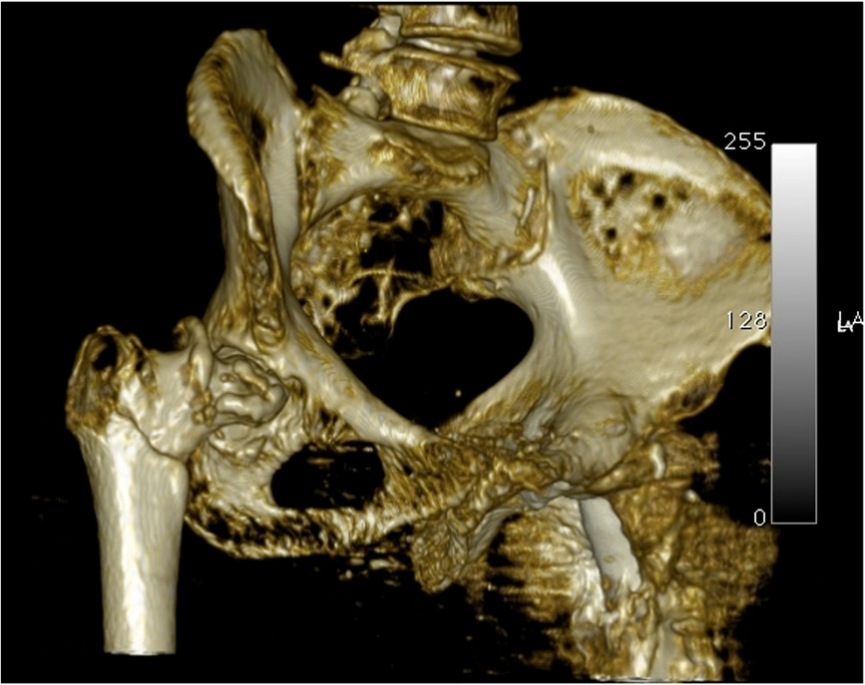
**Three-dimensional computed tomography scan of her right hip showing a simple intertrochanteric fracture associated with femoral head stage VI avascular necrosis, with the femoral head impacted in the acetabular rim.**

**Figure 4:**
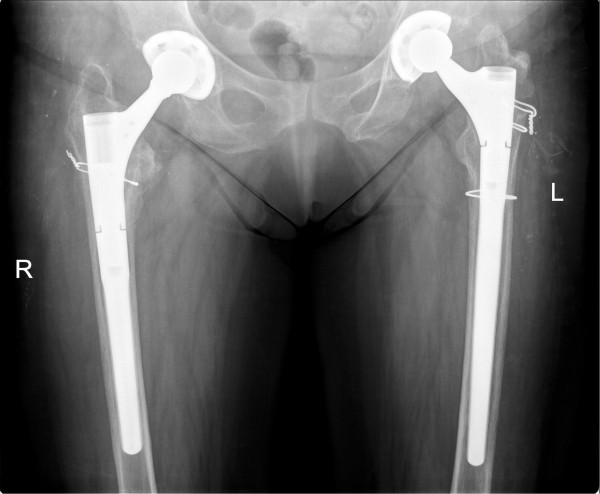
**Postoperative X-ray at one year postoperative for her right hip (two years for her left hip) showing bilateral primary total hip arthroplasty with uncemented acetabular shell and modular femoral components with radiographic union for both pertrochanteric fractures.**

A pelvic three-dimensional computed tomography scan (Figure 3) was performed to investigate the amount of acetabular erosion and the true diaphyseal extent of the fracture. After routine preoperative preparation it was decided that a primary THA would be the best indication to treat her right hip. Taking into account the operative and clinical success of the previous surgery, the small amount of acetabular destruction and the extension of the fracture line to just below the level of the lesser trochanter, the same implants were selected as for the previous surgery, apart from the component sizes (Figure 4). Antegrade nailing was used to treat the humeral fracture. Cerclage wiring was used in both surgeries to fix the fractured parts of the trochanter to the femoral stem. This has been shown to increase the overall strength of the implant-bone construct in fracture and femur osteotomy cases [8–10].

In the clinical setting of this particular case we feel that both hips had a strong indication for a primary THA with an uncemented modular femoral stem. On her left hip the trochanteric fracture was associated with hip OA (Figure 1). She reported a history of moderate anterior hip pain that was ongoing for about five months previous which was aggravated by prolonged walking and standing. Accounting for her previous degenerative symptoms and the trochanteric fracture, a one-stage primary THA was performed with the modular femoral stem for diaphyseal support. On her right hip the associated AVN with secondary OA and the extension of the fracture line below the level of the lesser trochanter made osteosynthesis undesirable. The advantages of the modular system include a wide range of proximal and distal components allowing for both metaphyseal and diaphyseal support combined with the advantages of an uncemented stem. At three months postoperatively, radiographic union was documented (Figure 4) and she could walk unassisted. Her Harris Hip Score was 68 and her WOMAC (Western Ontario and McMaster Universities Osteoarthritis Index) score was 64.8. At one-year postoperatively, she was ambulant, with a Harris Hip Score of 84 and a WOMAC score of 81.1.

On her left hip an osteolysis line can be observed at the level of the proximal cerclage wire with the proximal migration of the greater trochanter, caused by a non-union at this level. Tension banding has been reported to have good results for the fixation of trochanteric fractures and osteotomies [11]. Trochanteric nonunion is a known complication of THA with a modular femoral stem, and also one that has minor adverse effects on the final result [12]. In our case it caused a mild, painless, Trendelenburg gait with associated mild abductor weakness. The possibility of a trochanteric claw plate fixation was presented [13–15] but her choice was to continue with conservative treatment.

## Discussion

There was a previous belief in orthopedic practice that hip OA has a protective role towards fractures of the proximal femur. However a recent study by Calderazzi *et al*. determined no relationship between the severity of hip OA and the presence of a proximal femoral fracture [16]. Even more, the paper showed that while it does not have a protective role, the presence of OA increases the likelihood of a trochanteric fracture. These findings support those of Robstad *et al*. who compared 349 patients with proximal femoral fractures to a control group of 112 patients with hip contusion and found no significant difference in the rate of associated OA; they recorded more trochanteric fractures in patients with OA [17]. There is still no consensus in the literature regarding the inverse relationship between hip OA and proximal femoral fractures, with papers such as that of Franklin *et al*. supporting a one-third reduction of hip fractures in patients with OA [18].

Typical treatment of trochanteric fractures involves osteosynthesis with a SHS or a CMD for stable fractures, while unstable fractures are treated with a CMD which has a theoretical advantage in these fracture patterns [19, 20]. Hip replacement is regularly reserved for intracapsular fractures of the proximal femur in elderly patients due to their limited healing capability, and as a salvage procedure for extracapsular fractures with a failed fixation [7, 21]. However, there are a growing number of indications for a primary THA for extracapsular fractures such as advanced osteoporosis, severe comminution or association with hip OA. The main benefit of an accurately indicated and technically impeccable THA on such a patient would be the early mobilization and thus the low incidence of comorbidities such as decubitus sores, pulmonary infection, pulmonary atelectasis, deep vein thrombosis (DVT) or pulmonary embolism [22].

The increased morbidity and mortality rate, as high as 36% at one year post-surgery, the association of medical illness and the increased economic burden that these patients put on healthcare systems creates a necessity for implants that can assure a rapid return to the pre-injury level of activity [4]. THA is not supported unanimously in the literature, indeed Bonnaire *et al*. consider that while it is a potential option for patients with associated hip OA, for more comminuted fractures implanting a femoral component with a good offset without a varus or rotational failure will be increasingly difficult [2]. As THA has become a more ubiquitous surgery, the indication of primary THA can be broadened to include certain extracapsular proximal femoral fractures, particularly when they are associated with acetabular pathology, either degenerative or acute.

Modular femoral components were first developed for reconstructive purposes with indications in tumoral reconstruction or for revisions of a failed primary THA [23]. A paper by Weiss *et al*. reported the successful conversion of failed hip internal fixations (both SHS and CMD, the latter predominantly) on 30 patients in the Swedish Arthroplasty Registry between 2002 and 2009, with a 96% survival rate at three years and seven patients that needed further surgery [7]. Their increased rate of failure for CMD devices is consistent with data from other papers [5, 19, 20, 24]. Pui *et al*. concluded that prior fixation with CMD may be associated with significantly higher complication rates during conversion [25]. These revision procedures were associated with longer surgical times, increased complication rates and higher blood loss. THA has been advocated as a viable solution for patients that have sustained previous acetabular fractures. Further indications are the prevention of a revision surgery in old or highly osteoporotic patients, prevention of implant failure or difficult conversion surgeries, as described for CMD, and the possibility to address more difficult cases. For this reason elderly patients with increased comminution, damaged articular cartilage, impaction of the femoral head or acetabular destruction in the weight-bearing region of the acetabulum are best treated with a primary THA [6, 21, 22, 26, 27].

## Conclusions

While there are no large randomized studies to support this evidence, the work of several authors as well as our own clinical experience shows that internal fixation is not the only treatment option in patients with extracapsular hip fractures. Primary THA is a good indication in selected cases with associated pathology such as advanced OA, acetabular or femoral head deformities (previous fractures and/or avascular femoral head necrosis) allowing the avoidance of unnecessary intra and postoperative complications and a faster recovery, with higher postoperative scores. Uncemented modular femoral components have a good indication for these fractures because their diaphyseal area of support is distal to the fracture site, and because of their modularity that can benefit a wide array of trochanteric shapes, thus restoring the anatomical offset of the femur. The apposition of the fractured fragments around the stem is made intraoperatively and cerclage or cable osteosynthesis is used to maintain the fractured fragments reduced.

## Consent

Written informed consent was obtained from the patient for publication of this case report and accompanying images. A copy of the written consent is available for review by the Editor-in-Chief of this journal.

## Abbreviations

AP: Anteroposterior
AVN: Avascular Necrosis
CMD: Cephalomedullary Device
DVT: Deep Vein Thrombosis
OA: Osteoarthritis
ROM: Range of Motion
SHS: Sliding Hip Screw
THA: Total Hip Arthroplasty
WOMAC: Western Ontario and McMaster Universities Osteoarthritis Index.
